# Effects of shade stress on turfgrasses morphophysiology and rhizosphere soil bacterial communities

**DOI:** 10.1186/s12870-020-2300-2

**Published:** 2020-03-02

**Authors:** Juanjuan Fu, Yilan Luo, Pengyue Sun, Jinzhu Gao, Donghao Zhao, Peizhi Yang, Tianming Hu

**Affiliations:** Department of Grassland Science, College of Grassland Agriculture, Northwest A&F University, Yangling, 712100 Shaanxi China

**Keywords:** Community structure and diversity, Plant-soil feedback, Rhizosphere bacteria, Shade stress, Shade tolerance, 16S rRNA gene sequencing

## Abstract

**Background:**

The shade represents one of the major environmental limitations for turfgrass growth. Shade influences plant growth and alters plant metabolism, yet little is known about how shade affects the structure of rhizosphere soil microbial communities and the role of soil microorganisms in plant shade responses. In this study, a glasshouse experiment was conducted to examine the impact of shade on the growth and photosynthetic capacity of two contrasting shade-tolerant turfgrasses, shade-tolerant dwarf lilyturf (*Ophiopogon japonicus*, OJ) and shade-intolerant perennial turf-type ryegrass (*Lolium perenne*, LP). We also examined soil-plant feedback effects on shade tolerance in the two turfgrass genotypes. The composition of the soil bacterial community was assayed using high-throughput sequencing.

**Results:**

OJ maintained higher photosynthetic capacity and root growth than LP under shade stress, thus OJ was found to be more shade-tolerant than LP. Shade-intolerant LP responded better to both shade and soil microbes than shade-tolerant OJ. The shade and live soil decreased LP growth, but increased biomass allocation to shoots in the live soil. The plant shade response index of LP is higher in live soil than sterile soil, driven by weakened soil-plant feedback under shade stress. In contrast, there was no difference in these values for OJ under similar shade and soil treatments. Shade stress had little impact on the diversity of the OJ and the LP bacterial communities, but instead impacted their composition. The OJ soil bacterial communities were mostly composed of *Proteobacteria* and *Acidobacteria*. Further pairwise fitting analysis showed that a positive correlation of shade-tolerance in two turfgrasses and their bacterial community compositions. Several soil properties (NO_3_^−^-N, NH_4_^+^-N, AK) showed a tight coupling with several major bacterial communities under shade stress. Moreover, OJ shared core bacterial taxa known to promote plant growth and confer tolerance to shade stress, which suggests common principles underpinning OJ-microbe interactions.

**Conclusion:**

Soil microorganisms mediate plant responses to shade stress via plant-soil feedback and shade-induced change in the rhizosphere soil bacterial community structure for OJ and LP plants. These findings emphasize the importance of understanding plant-soil interactions and their role in the mechanisms underlying shade tolerance in shade-tolerant turfgrasses.

## Background

Light provides energy for photosynthesis, modulating plant growth, development, and morphogenesis [1]. Shade stress adversely impacts chlorophyll content, chloroplast ultrastructure, photosynthetic processes, and plant morphology [2–5]. Urban greening currently employs a combination of trees, shrubs and grass, resulting in an increase in shaded lawn area. As a result, shade stress presents a major challenge to turf grass growth in urban environments. It has been estimated that 20–25% of all turf grass in the USA [6], and 50% of turf grass in China, are subjected to varying degrees of shade [7]. To maintain carbon gain, plants adjust their morphological and physiological characteristics in response to shade [7, 8]. For example, to maintain carbon gain plants can increase elongation growth, specific leaf area, and biomass allocation to leaves [8–12]. They can also reallocate nutrients from roots to leaves, and increase the input of photosynthetic enzymes to maximize photosynthesis under shade stress [12, 13].

Shade stress also leads to lower air flow and directly alters soil humidity and temperature. These alterations change the physicochemical properties of soil, creating microclimates that influence soil microbial structure and diversity [14]. Soil microbial communities may alter under shade stress due to selectively gathering tolerant microbial groups, for example shade tolerant *Bauhinia variegata* had higher the Gram+: Gram- bacteria ratio than non-tolerant *B. brachycarpa* [12].

Numerous studies suggest that plants’ responses to abiotic stresses may be mediated by soil microorganisms [15, 16]. Soil microbial communities can directly impact plant growth and functional traits by altering defense and metabolic pathways [15, 17]; They can also indirectly impact plant performance by altering soil properties, which influence plant traits associated with resource acquisition and use [18]. A recent study comparing plants grown in sterile and non-sterile soils demonstrated soil-plant feedback effects such as the importance of soil microorganisms for plant traits and plant tolerance to shade stress [12]. Dahl et al. [19] also pointed out that warming and shading changed fungal community composition in Arctic soils. Although these studies have established important roles for soil microbial communities in influencing plants under shade stress, there is little understanding of the interplay between turfgrasses and their soil microbial communities under shade conditions.

The root architecture in soil reflects a plant’s ecological adaptation and may increase plant survival under stress [20]. Studies have indicated that root phenotypic traits can potentially influence the root and the rhizospheric soil microbiome [21, 22]. Saleem et al. [23] also showed that the root system architecture filters and recruits different microbial communities. However, there is little understanding of how shade influences root morphology and root-associated bacterial communities in turfgrasses.

Dwarf lilyturf (*Ophiopogon japonicus* (Linn. f.) Ker-Gawl, OJ) is an important green plant in the Liliaceae family with strong inherited shade tolerance. *Lolium perenne* (LP), is one of the most important shade-intolerant cool-season perennial turf-type ryegrasses in temperate climates. In this study, we investigated the effects of shade on the photosynthetic capability and the rhizosphere soil bacterial communities of OJ and LP plants grown in the same lawn under different light conditions. We also analyzed soil-plant feedback effects in both plant species. The following hypotheses were tested: i) shade tolerance is modified by soil microorganisms as a result of soil feedback effects; ii) the differing shade-tolerances of OJ and LP correlate with altered soil bacterial community compositions; iii) the altered rhizosphere bacterial community structure observed under shade stress is caused by shade-induced changes in soil chemistry and differing plant photosynthetic capabilities.

## Results

### OJ and LP seedling growth and plant traits

**Fig. 1:**
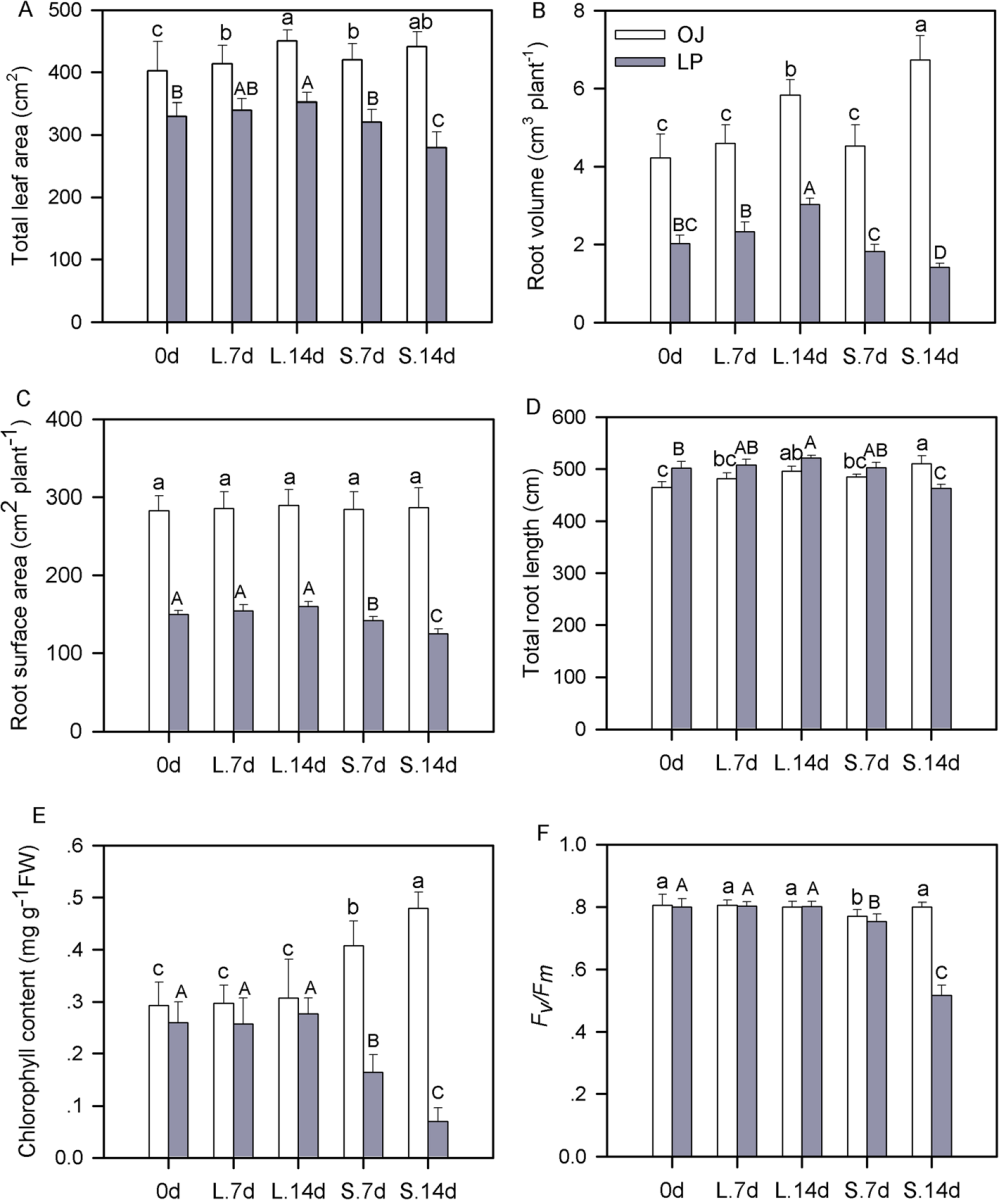
Plant total leaf area (**a**), total root length (**b**), root surface area (**c**), and root volume (**d**), chlorophyll content (**e**), and chlorophyll fluorescence (*F*_*v*_*/F*_*m*_) (**f**) of shade-tolerant OJ (*Ophiopogon japonicus*) and shade-intolerant LP (*Lolium perenne*) under shade stress. Values are the means ± SD of six pot replicates. Lowercase or capital letters indicate significant differences (*P* < 0.05, LSD test) in OJ or LP

OJ and LP seedlings were exposed to shade stress and examined to determine their growth response to this stress (Fig. 1, Additional file 1: Table S1). Shade treatment resulted in different growth suppression in the two plants. Shade did not significantly influence OJ leaf area but resulted in a 14.9% decrease (*P* < 0.05) in LP leaf area when leaves were exposed to 14 d of shade stress compared to a non-shade control (Fig. 1a). Shade treatment significantly decreased (*P* < 0.01) total LP root length, root surface area, and root volume, while OJ exhibited superior acclimation to shade stress (Fig. 1b-d). In addition, OJ and LP had different changes in chlorophyll content in response to shade stress. Shade stress increased OJ chlorophyll content, while shade stress reduced LP chlorophyll content (Fig. 1e). Fluorescence parameters (*F*_*v*_*/F*_*m*_) for LP chlorophyll *a* were reduced significantly compared with OJ chlorophyll *a*, indicating that OJ maintained higher photosynthetic capacity under shade stress (Fig. 1f). These results demonstrate that OJ is more shade-tolerant than LP.

**Fig. 2:**
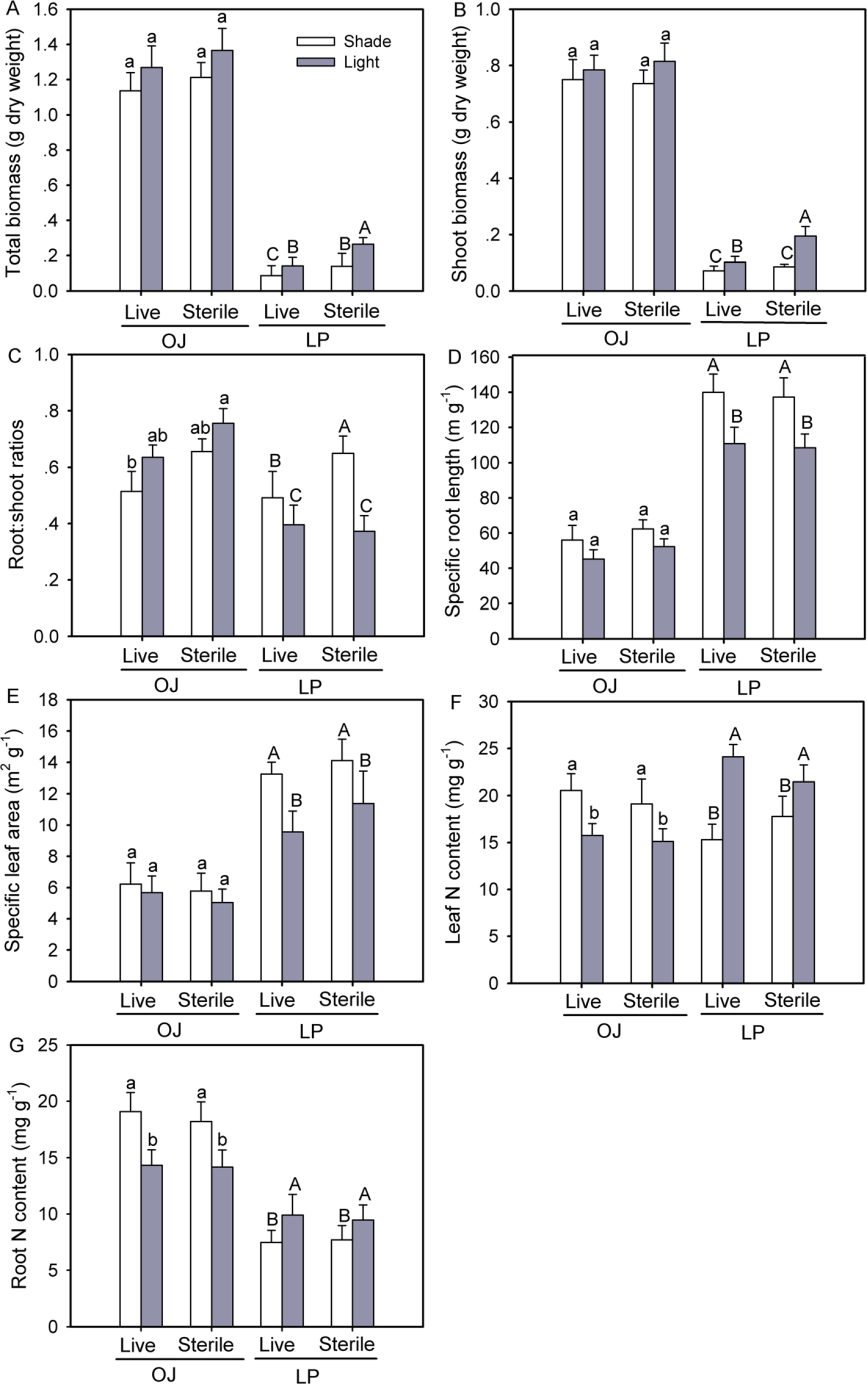
Effects of shade and soil treatments on (**a**) total biomass, (**b**) shoot biomass, (**c**) root:shoot ratios, (**d**) specific stem length, (**e**) specific leaf area, (**f**) leaf N content, and (**g**) root N content of OJ (*Ophiopogon japonicus*) and LP (*Lolium perenne*). Values are the means±SD of six replicates. Lowercase or capital letters indicate significant differences (*P* < 0.05, LSD test) in OJ or LP

Shade-intolerant LP was more responsive than shade-tolerant OJ to shade and changes in the soil microbial community (Fig. 2; Additional file 2: Table S2). Shade stress negatively affected total LP plant biomass during the experimental period, but no change was observed in OJ (Fig. 2a, b). The total LP dry and shoot biomasses were lower in live soil compared to those in sterile soil. This occurred under non-shade and shade stress. In contrast, no significant difference was observed in OJ under the same conditions.

For LP, root: shoot ratios were higher in the shade compared to non-shade conditions. LP also exhibited higher ratios in sterile compared to live soil under shade stress, however there was no significant difference in light response between live and sterile soil (Fig. 2c). Shade significantly increased (*P* < 0.05) LP specific root length, but we observed no significant change between sterile and live soil. We did observe a slight difference in OJ specific root length under light and soil treatments (Fig. 2d).

As observed with specific root length, LP specific leaf area responded to shade treatment, with higher values (*P* < 0.05) in shade compared to non-shade (Fig. 2e). Plant leaf and root N content showed significant responses (*P* < 0.05) to shade treatment. The synergistic effect of shade treatment and the presence of soil microbes on root and leaf N was not significant (Fig. 2f, g; Additional file 2: Table S2). Compared to non-shade treatment, shaded plants in sterile soil had a 17.18% decrease in leaf N content and shaded plants in live soil displayed a 36.52% decrease in leaf N content. The LP root N content decreased 18.73 and 24.57% in shaded versus non-shaded LP plants under sterile and live soil treatments, respectively. For OJ, shade increased the N content in live soil: 30.61% in leaves and 33.01% in roots. The corresponding values for shaded sterile soil were 26.58 and 28.45% for leaves and roots, respectively (Fig. 2f, g).

**Fig. 3:**
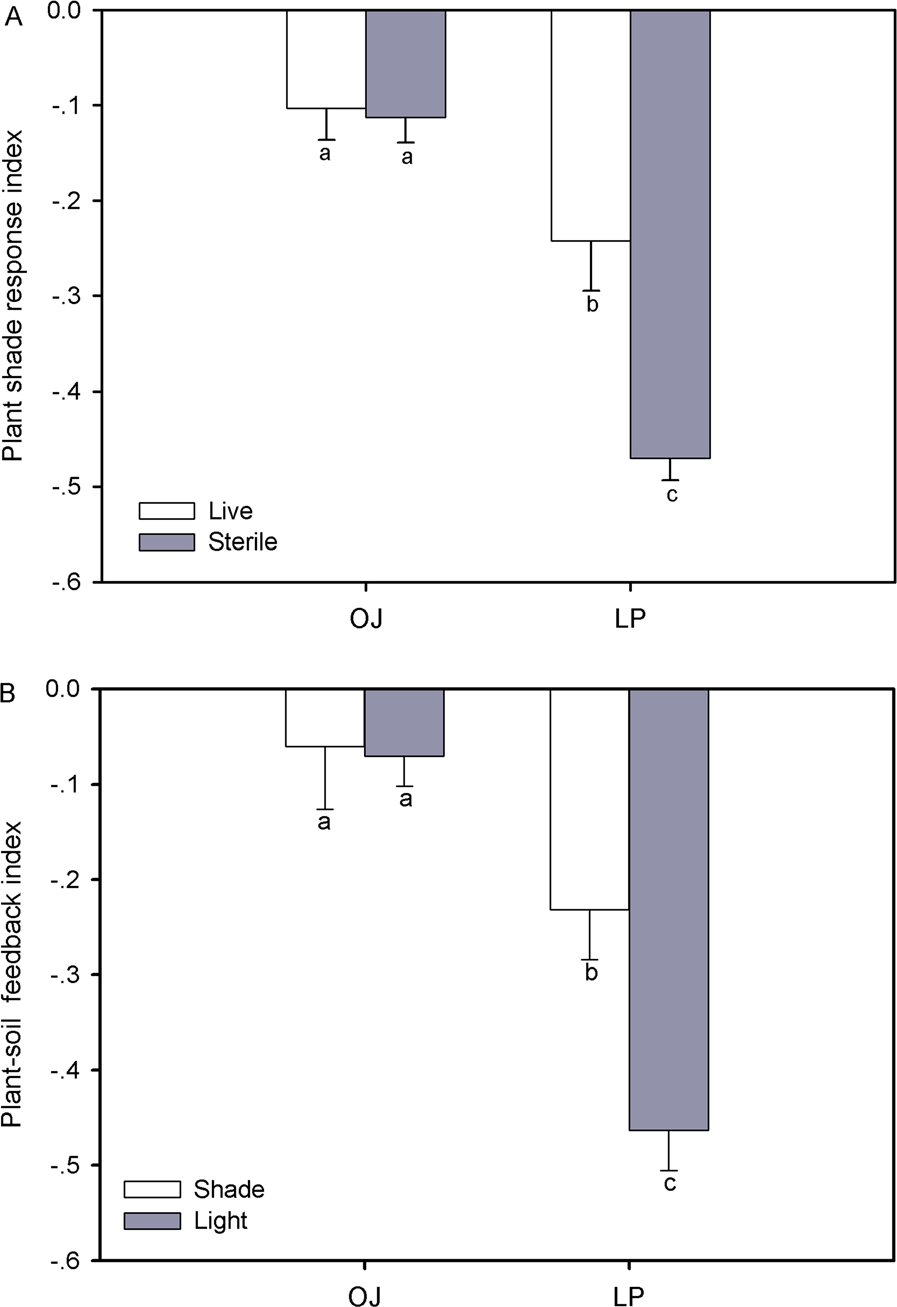
Plant shade response index (**a**) in live or sterile soil treatments and (**b**) soil-plant feedback index under highlight and shade conditions. Indices are based on seedling biomass of OJ (*Ophiopogon japonicus*) and LP (*Lolium perenne*). Values are the means±SD of six replicates. Lowercase or capital letters indicate significant differences (*P* < 0.05, LSD test) in OJ or LP

Soil treatment and species interaction significantly affected the plant shade response index (Fig. 3a; Additional file 3: Table S3). The plant shade response index of LP responded to soil treatments with significantly higher values (*P* < 0.05) in live soil versus sterile soil, while no difference in OJ plant shade response index was observed (Fig. 3a). Also, shade and species interaction had a significant effect on soil-plant feedback index (Fig. 3b; Additional file 3: Table S3). The LP soil-plant feedback index was consistently lower (*P* < 0.05) in shade compared to non-shade treatments, but there was no difference in the OJ soil-plant feedback value between non-shade and shade treatments (Fig. 3b).

### Soil chemical characteristics

Shade stress significantly influenced most of the physicochemical properties analyzed (Table 1). Both OJ and LP soil showed significant increases in the NO_3_^−^-N content with shade treatment (*P* < 0.001). Conversely, shade treatment decreased TP, TK and AK in both soil types compared to non-shade treatment, with a greater effect with LP soil. Shade treatment of OJ resulted in a significant decrease in rhizosphere NH_4_^+^-N and a significant increase in the rhizosphere AP content. The opposite trend was observed with LP. Shade treatment had a small effect on the soil TN, SOC, C:N ratio, and rhizosphere pH level.

**Table 1:** Responses of soil physicochemical variables to shade treatment. Values are the means (SD) of six pot replicates. Different lowercase or capital letters indicate significant differences (*P* < 0.05, LSD test) in OJ (*Ophiopogon japonicus*, shade tolerant) or LP (*Lolium perenne*, shade-intolerant) soil. ***P < 0.001, **P < 0.01, *P < 0.05

Soil sample	Total N (g kg^− 1^)	Organic C (g kg^− 1^)	Soil C:N	Total P (g kg^− 1^)	Total K (g kg^− 1^)	NH_4_^+^-N (mg kg^− 1^)	NO_3_^—^N (mg kg^− 1^)	Available P (mg kg^− 1^)	Available K (mg kg^− 1^)	PH
OJ.0d	1.07(0.23)	24.38(0.27)	22.79(1.79)	0.52(0.045)b	17.19(0.19)b	6.99(0.026)a	2.08(0.026)d	0.40(0.014)b	140.35(11.92)	7.46(0.029)
OJ.L.7d	1.18(0.27)	27.43(3.16)	23.25(2.53)	0.62(0.13)a	18.17(0.18)a	2.93(0.27)b	4.73(0.38)cd	0.57(0.043)b	184.79(32.29)	7.38(0.025)
OJ.L.14d	1.19(0.19)	24.21(1.97)	20.34(1.70)	0.58(0.035)a	18.14(0.12)a	2.91(0.61)b	7.79(2.12)b	0.47(0.024)b	162.79(22.92)	7.41(0.092)
OJ.S.7d	1.08(0.14)	21.97(4.04)	20.34(1.38)	0.51(0.05)b	17.83(0.36)a	2.15(0.89)b	5.84(0.53)bc	1.16(0.033)a	162.02(36.03)	7.67(0.089)
OJ.S.14d	1.09(0.16)	22.13(3.62)	20.30(1.21)	0.57(0.040)a	16.98(0.20)b	2.63(0.26)b	11.64(2.59)a	1.47(0.024)a	136.83(6.09)	7.36(0.061)
LP.0d	1.24(0.09)	25.36(0.87)	20.45(0.66)	0.54(0.032)b	16.88(0.13)bc	4.78(0.96)b	0.84(0.15)d	3.56(0.49)a	176.01(5.61)	7.51(0.085)
LP.L.7d	1.21(0.02)	21.88(0.74)	18.08(0.73)	0.68(0.11)a	19.18(0.96)a	4.67(0.47)b	3.17(0.201)c	2.33(0.85)b	153.59(15.53)	7.49(0.093)
LP.L.14d	1.17(0.15)	24.99(1.27)	21.36(1.76)	0.60(0.021)ab	17.36(0.41)b	5.94(1.44)a	4.28(0.092)b	3.30(1.02)a	177.69(41.05)	7.39(0.13)
LP.S.7d	1.12(0.10)	22.97(3.19)	20.51(1.09)	0.59(0.015)ab	17.56(0.38)b	5.08(0.62)ab	5.12(0.25)b	3.03(0.46)a	143.13(8.82)	7.42(0.25)
LP.S.14d	1.06(0.05)	21.95(2.79)	20.51(2.50)	0.56(0.015)b	15.72(0.04)c	6.23(0.43)a	6.55(0.76)a	0.68(0.036)c	142.67(3.16)	7.65(0.061)
F value for ANOVA										
Treatments (T)	0.504	0.919	0.738	3.279^*^	12.394^***^	4.302^*^	37.691^***^	1.910^*^	1.879	1.562
Species (S)	0.328	0.105	1.885	2.430	0.310	22.301^***^	35.171^***^	0.114^*^	0.023	0.545
T × S	0.346	0.936	2.158	0.393	8.531^***^	7.487^**^	4.009^***^	1.010^*^	2.100	4.774^*^

### Bacterial diversity and community composition response to shade stress

Amplicon products of the V4 region of the 16S rRNA gene were obtained from each of the 60 samples and sequenced using the Illumina HiSeq 2500 platform. A total of 5,371,314 bacterial clean reads were obtained. These sequences were grouped into 11,485 OTUs according to a 97% similarity threshold. The rarefaction curves (Additional file 4: Figure S1) demonstrated that the sequencing depth in these samples was sufficient to cover the full diversity.

**Fig. 4:**
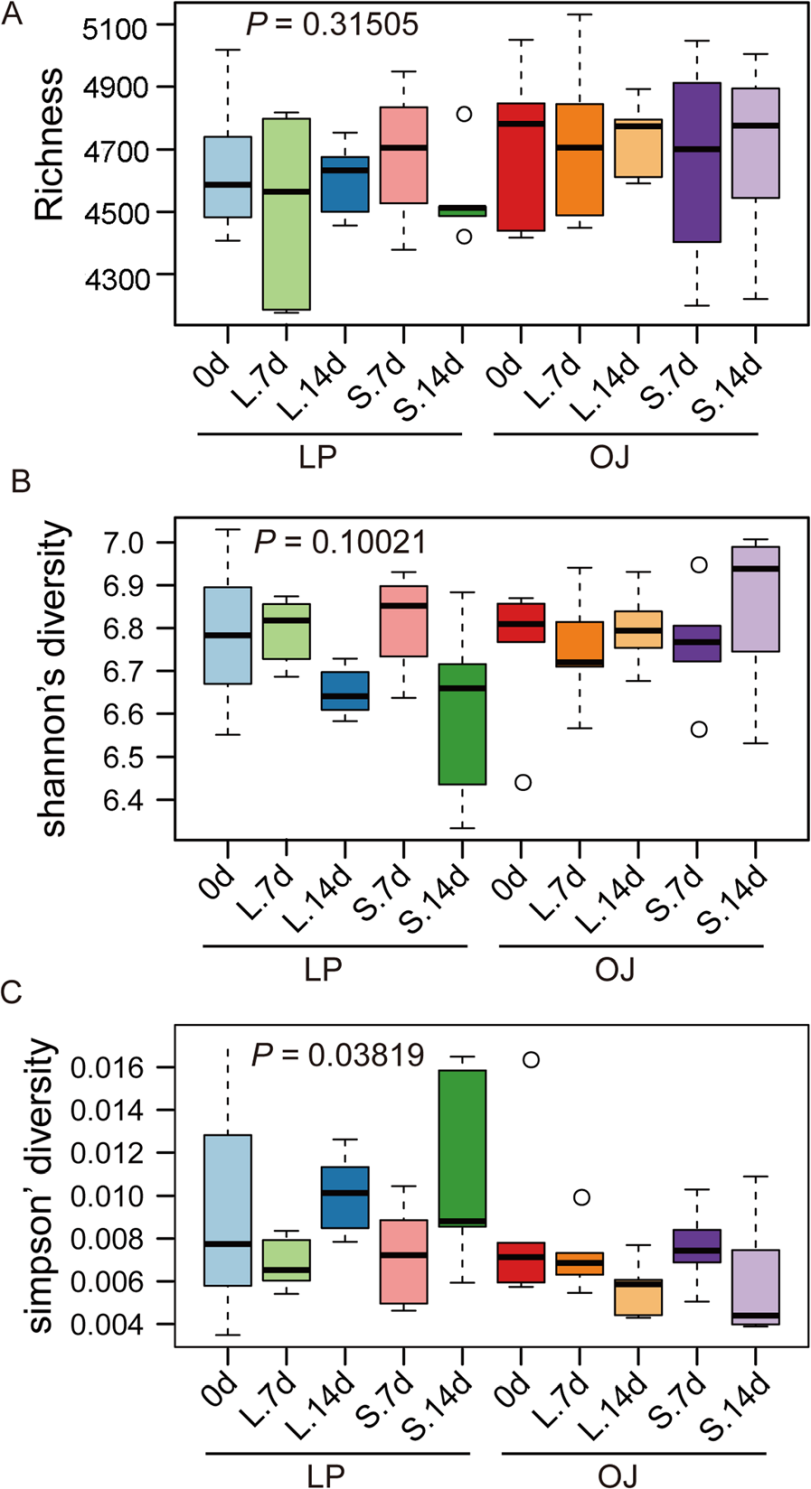
Boxplots of Richness (**a**), Shannon’s diversity index (**b**) and Simpson’s Evenness (**c**)] of bacterial communities based on OTUs defined at 97% sequence similarity. Black dots represent soil samples outliers

The OJ and LP rhizosphere soil bacterial communities did not have similar alpha diversity features, as measured by the OTU richness, Shannon’s diversity index (H) and Simpson’s Evenness (E) (Fig. 4). The OTU richness and diversity did not show significant differences between the two rhizosphere soils. However, the evenness increased (*P* < 0.05) in OJ soil under shade stress but decreased in LP soil. This suggests that a few numerically dominant OTUs inhabit the LP rhizosphere.

**Fig. 5:**
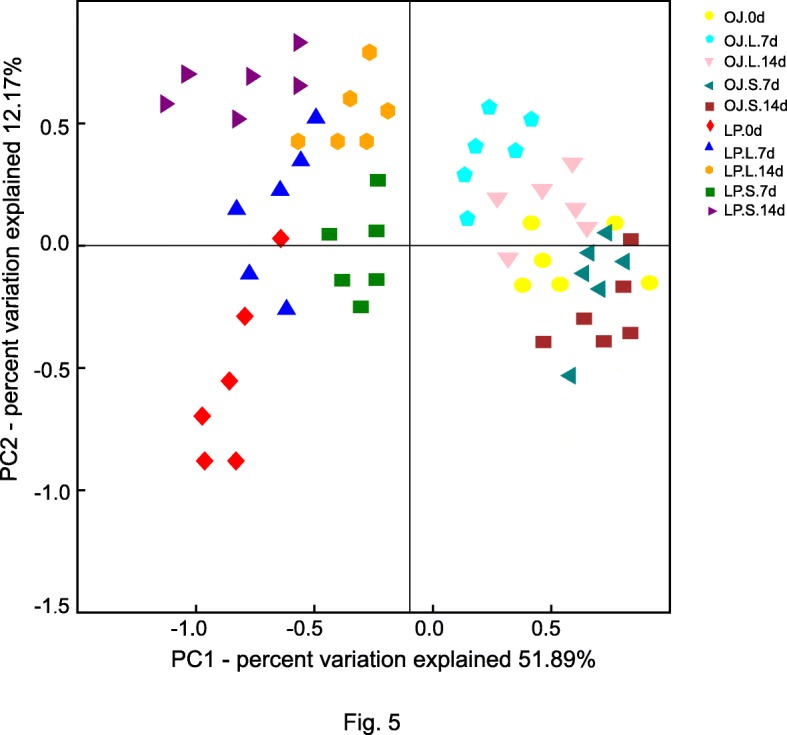
Principal coordinates analysis (PCoA) based on Bray-Curtis distances between rhizosphere soil bacterial communities in shade-tolerant OJ and shade-intolerant LP under shade stress

The bacterial community composition between shade treatments in OJ and LP soils were analyzed using PCoA based on Bray-Curtis dissimilarity. The PCoA analysis explained 64.06% of variation (two axes) in bacterial community composition. Shade treatments led to a distinct bacterial community structure (PERMANOVA, *P* < 0.05), and the bacterial community structures of the OJ and LP rhizosphere soils were also obviously different (Fig. 5). Further evidence showed that the bacterial communities collected within the OJ rhizosphere on the one hand, and LP rhizosphere on the other, overlapped partially in the PCA plot (Additional file 5: Figure S2), indicating that OJ and LP soils had different bacterial community structures.

**Fig. 6:**
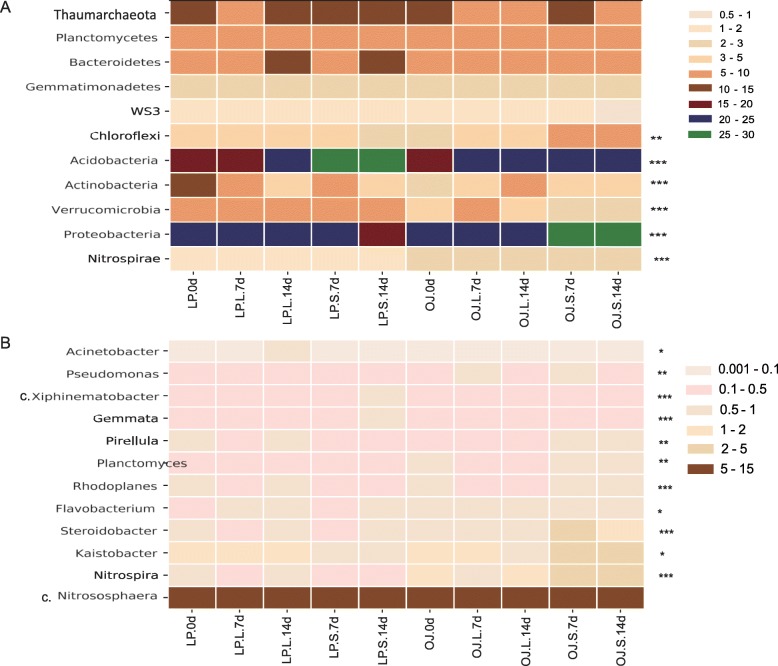
Changes in the bacterial community composition at the phylum level (relative abundance > 1%, **a**), and genus level (relative abundance > 0.5%, **b**) under shade stress. Asterisks indicate statistically significant differences according to Kruskal-Wallis tests (*P < 0.05; **P < 0.01 and ***P < 0.001)

In both OJ and LP rhizospheric soil, the edaphic bacterial communities harbored 11 different phyla (accounting for more than 93% in each sample). The most numerically dominant phyla were *Proteobacteria* followed by *Acidobacteria* and *Thaumarchaeota* (Fig. 6a). *Proteobacteria*, *Actinobacteria*, and *Chloroflexi* decreased in LP soil in response to shade stress, but an increase or a lower degree of change was observed in shaded OJ soil. In contrast, shade led to higher abundances of *Verrucomicrobia* and *Acidobacteria* in LP soil, compared to OJ soil under shade stress (Kruskal-Wallis, *P* < 0.01).

There were 12 genera (> 0.5%) within the classes *Alpha* and *Gamma Proteobacteria*, *Flavobacteria*, *Planctomycetia*, *Spartobacteria*, *Nitrospira*, and *Thaumarchaeota*. The genus *Candidatus Nitrososphaera* was clearly dominant within the taxonomic structure of the bacterial community (Fig. 6b). The most evident differences between OJ and LP rhizosphere soil bacterial communities were the opposing trends in the abundance of *Nitrospira*, *Steroidobacter*, *Kaistobacter* and *Pirellula*. These genera were unchanged or increased with increasing shade treatment in OJ soil, but they tended to decrease in LP soil. In contrast, the relative abundance of *Rhodoplanes*, *Planctomyces*, and *Pseudomonas* was larger (Kruskal-Wallis, *P* < 0.01 or *P* < 0.001) in OJ soil under shade treatment, compared to LP soil. In LP soil *Gemmata* was more abundant than in OJ soil (Kruskal-Wallis, *P* < 0.001), although shade stress decreased the relative abundance of this genus in both soils.

### Core microbial players associated with rhizosphere soil in OJ and LP

The core bacteriome of OJ and LP rhizosphere soils was determined to examine shifts in the bacterial communities observed with the different host types. This analysis suggested that a specific taxonomy may exist which is particularly well adapted and prominent under different growth conditions. We found that OJ rhizosphere soil was dominated by OTUs identified as: *Nitrosovibrio* (19.1% of total core bacterial OTU), *Aquicella* (12.5%), *Planctomyces* (11.8%), *Pseudomonas* (11.2%), *Nitrospira* (10.3%), *Steroidobacter* (10.3%), *Flavobacterium* (8.8%), *Kaistobacter* (5.2%), *Bacillus* (6.8%), and *Rhodoplanes* (5.1%), which mostly belongs to *Proteobacteria*. *Nitroso vibrio tenuis* and *Candidates Nitrososphaera_SCA1145* (both 5.9%) were also identified in the OJ rhizosphere soil core (Additional file 6: Table S4). In contrast, the LP rhizosphere soil was dominated by *Acinetobacter* (21.0%), *Flavisolibacte* (19.3%), and *Skermanella* (17.1%) (belonging to *Proteobacteria* and *Bacteroidetes*, respectively).

### Relationships between shade-tolerant parameters and bacterial communities

**Fig. 7:**
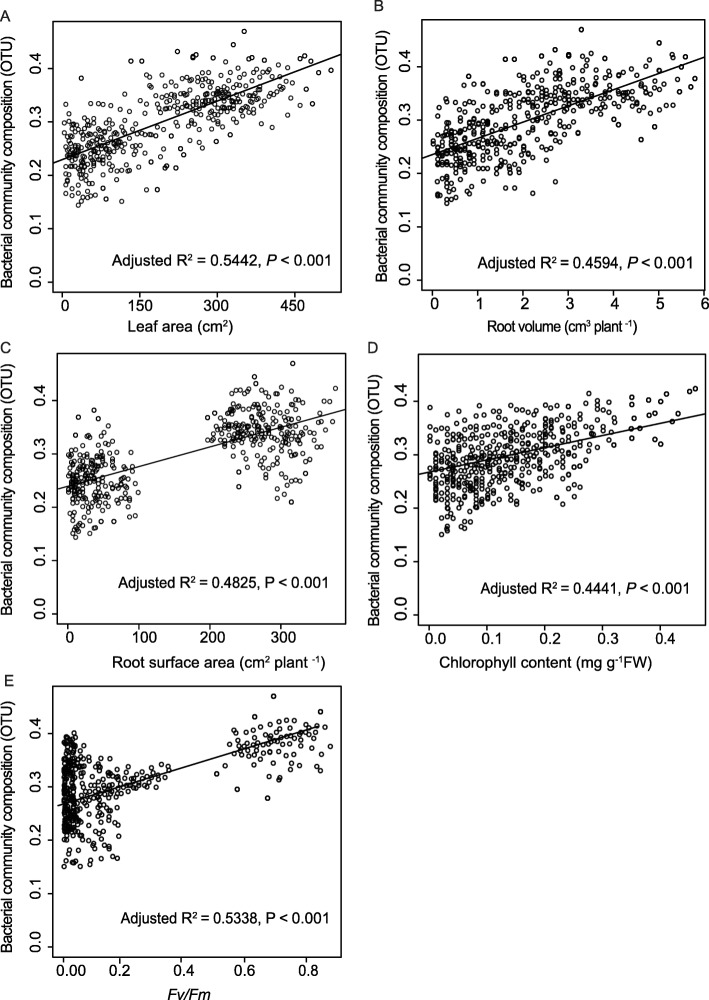
Relationships between total leaf area (**a**), root volume (**b**), root surface area (**c**), *F*_*v*_*/F*_*m*_ (**d**), and chlorophyll content (**e**) in bacterial community composition under shade stress. The relationship between total root length and bacterial community composition was not significant (data not shown). The plot shows the 95% confidence interval of the fit

There was a significant positive relationship between plant shade tolerance and soil bacterial community composition (Fig. 7). Among all the shade-tolerant indicators measured, leaf area, *F*_*v*_*/F*_*m*_, chlorophyll content, and root morphology were correlated with soil bacterial community composition (*P* < 0.001 for all).

### Relationships between bacterial community and environmental variables

**Fig. 8:**
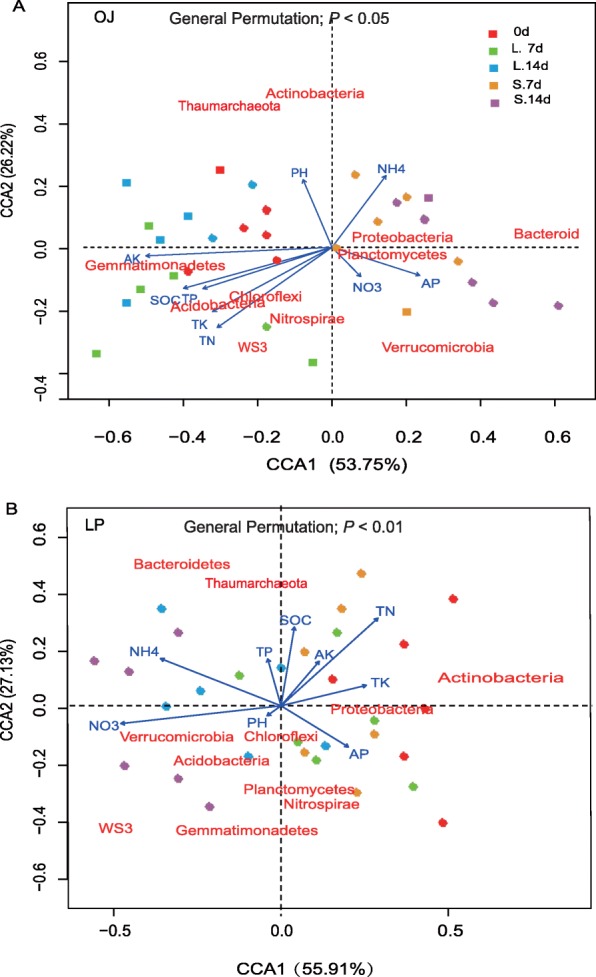
Canonical correspondence analysis (CCA) of bacterial communities based on Bray Curtis distances in rhizosphere soil bacterial communities of OJ (**a**) and LP (**b**) under shade stress. Arrows indicate the direction and magnitude of bacterial taxa associated with soil physicochemical characteristics. Permutation tests confirmed the effect of the soil factors as drivers of the bacterial community

The OJ and LP soil bacterial community structures displayed clear, individual correlations (*P* < 0.001 or *P* < 0.05) to soil physicochemical variables including NH_4_^+^-N, NO_3_^−^-N, and TK as shown by the Mantel test (Additional file 7: Table S5). CCA analysis revealed that the OJ and LP rhizosphere soil bacterial communities were affected differently by edaphic chemical parameters under the shade treatments examined. The proportion of total variability of OJ and LP soil bacterial communities attributed to the explanatory variables was 73.21 and 82.57%, respectively. This partition of variability was significant (general permutation test, *P* < 0.01 or 0.05; 999 replicates; Fig. 8; Additional file 8: Table S6). AK and total N were the major factors affecting the bacterial assemblages in OJ soil as judged by the length of the vectors shown in our CCA plots. In OJ soil, AK and total N were positively correlated (*P* < 0.05) with *Gemmatimonadetes*, *Chloroflexi*, *Acidobacteria*, *Nitrospirae*, and *WS3*. For OJ soils, CCA was consistent with the trends revealed by PCA showing a clear separation between control and shade treatment (Additional file 5: Figure S2). The TN, NO_3_^−^-N, and NH_4_^+^-N concentrations, three directly interlinked parameters, had a strong effect on bacterial assemblages in the LP soil. TN and NH_4_^+^-N were positively correlated (*P* < 0.05) with *Actinobacteria, Bacteroidetes*, and *Thaumarchaeota.* Taxa, such as *Verrucomicrobia*, *Chloroflexi*, *Acidobacteria*, *Planctomycetes*, *Gemmatimonadetes*, and *WS3* were positively correlated (*P* < 0.01) with NO_3_^−^-N. Additionally, shade treatments of different durations separately clustered in LP soil.

## Discussion

The current knowledge of the plant shade stress response has arisen from studies of plant physiology and morphology and has neglected the contribution of soil-plant interactions to shade tolerance. This work used two turfgrass genotypes with contrasting shade tolerances to investigate the plant growth response induced by shade and the role of soil microbial communities in this response.

### Physiological responses of OJ and LP plant to shade stress

We uncovered several differences between OJ and LP through analysis of the photosynthetic response and growth suppression that accompanied shade stress. Our physiochemical data demonstrated that LP exhibited more severe growth suppression due to shade stress than OJ. We observed a larger decline in leaf area, total root length, root volume, and surface area in LP versus OJ under shade stress. Similar results have been observed in several tree species, showing that shade-tolerant red oak had greater leaf area and dry mass than shade-intolerant species [9]. Plant photosystem II is sensitive to various environmental stresses, including shade stresses [24]. Chlorophyll *a* fluorescence (*F*_*v*_/*F*_*m*_) is a valuable indicator of stress tolerance [4, 25]. We analyzed these paramaters for LP and OJ and our results show that under shade stress OJ maintained higher *F*_*v*_/*F*_*m*_ and chlorophyll (*a* + *b*) content than LP, but no significant difference between LP and OJ was observed in sunlight. This suggests that OJ has a better photosynthetic capacity under shade stress than LP and thus OJ is more shade-tolerant than LP.

### Plant shade tolerance is mediated by soil-plant feedback

Given that soil microorganism could directly and indirectly affect most plant functional traits [12, 16, 18]. We analyzed the role of soil-plant feedback to plant shade tolerance to further clarify the shade tolerance mechanisms of OJ and LP. We found that shade stress reduced LP whole-plant biomass, and affected LP shoots and roots, however we did not observe this change with OJ. However, shade induced reducations in LP dry biomass that were significantly lower in live soil treatments compared to sterile soil. This resulted in a less negative plant shade response index in live soil treatments, consistent with the recent study in two *Bauhinia* tree species with contrasting shade tolerance [12].

The present data agrees with our first hypothesis that plant shade tolerance would be modified by soil microorganisms. LP plant biomass was lower in live soil treatments. This agreed with previous studies showing that soil microorganisms have negative feedback effects on plant growth [26, 27]. Soil microorganism-induced declines in LP biomass were accompanied by reductions in plant N content, indicating that soil microbes may compete with plants for nitrogen [28].

As with the interactions observed between soil microbes and shade on plant biomass, the interactive effects on soil-plant feedback was greater for LP compared to OJ. Shade-tolerant OJ plants are less sensitive to soil microbe-mediated soil-plant feedback due to a higher defense against soil pathogens and lower dependence on symbionts [29]. Also, both OJ and LP allocated more biomass to shoots in live soil compared to sterilized soil as shown by lower root: shoot ratios in live soil. This effect is an adaptive response to a limited light resource. Plants have the ability to adjust their traits and patterns of biomass allocation to capture limited resources and maintain carbon gain [30].

### Plant shade tolerance is related to bacterial community composition

The observed shade-induced changes in soil feedback were accompanied by a shift of soil bacterial community structure. In both OJ and LP we found that shade stress had little impact on bacterial richness and soil community diversity. This is consistent with other studies showing that community diversity is not significantly impacted by drought [31, 32]. Similar results have been reported in salt stress, demonstrating that increasing salinity has no effect on total bacterial community richness [33]. The observed shifts in the soil microbiome when OJ and LP were shade stressed involved changes in relative bacterial abundance, rather than outright abolition of shade susceptible taxa and concomitant appearance of tolerant ones. This helps explain the lack of change in alpha-diversity.

The rhizosphere soils of OJ and LP plant with various shade treatments had significantly different bacterial community compositions. The *Proteobacteria* and *Actinobacteria* have been shown to accumulate in OJ soil in response to shade stress, while they have been shown to decrease in LP soil. Prior studies have shown that *Proteobacteria* and *Actinobacteria* response to various environmental stresses, such as drought, salt and heavy metal stresses [34–37]. *Actinobacteria* are implicated in promoting plant growth under stress [38]. Many of them are known to form spores, which are resistant to adversity and can survive under stress conditions [39, 40].

Alpha and gamma *Proteobacteria* play a vital role in OJ soil in response to shade stress, as indicated by greater increase in genera of *Kaistobacter*, *Steroidobacter*, *Pseudomonas* observed in OJ soil. Inoculants of plant growth-promoting rhizobacteria (PGPRs) genera such as *Pseudomonas*, *Flavobacterium*, and *Arthrobacter* have been shown to ameliorate stress in plants by mediating a variety of physiological and biochemical changes [41–44]. Our study shows that species such as *Nitrosovibrio_tenuis*, *Reyranella_massiliensis*, *Arthrobacter_psychrolactophilus*, and *Flavobacterium_succinicans*, which belong to phyla of *Proteobacteria, Actinobacteria and Bacteroodetes*, are more abundant in OJ soil. This suggests that OJ prefer these genera and they may be markers of better shade-tolerance.

To further clarify this assumption and study the correlation of shade-tolerance parameters and bacterial community composition, pairwise fitting analyses were performed to compare above and below-ground morphology, photosynthetic capacity and bacterial community composition. We found that the leaf area, root volume, surface area, *F*_*v*_*/F*_*m*_, and chlorophyll (*a* + *b*) content were positively and significantly related to soil bacterial community composition. This observation is in line with our second hypothesis, and with the last part of our third hypothesis. A few studies have also found that root phenotypic traits or architecture potentially influence rhizosphere and root microbial communities in *Phaseolus vulgaris* and *Nicotiana* species [22, 23]. Similar observations have been shown in wild and domesticated *Phaseolus vulgaris*, where the divergence in rhizobacterial community composition between wild and modern bean accessions is associated with differences in specific root length [21].

### Changes in soil physicochemical properties play an essential role in shaping bacterial communities under shade stress

Soil acts as a strong ecological filter affecting the bacterial community structure and diversity. Numerous studies of microbial communities under abiotic stress have shown that soil factors govern microbial community structure [45–47]. Bottomley et al. [48] observed that soil NH_4_^+^-N was a dominant environmental factor that influenced bacterial community structures. This is due to the fact that soil NH_4_^+^-N is the main nitrogen source for bacteria as seen by ^15^N isotope tracing [49]. Similarly, Nguyen et al. [50] also reported that cotton field soil bacterial diversity and composition were related to soil NH_4_^+^-N and total N content exposed to post-waterlogging or post-prolonged drought. Consistent with our third hypothesis, AK and total N were the major drivers in OJ rhizosphere soil and they were positively associated with *Gemmatimonadetes*, *Chloroflexi*, *Acidobacteria*, *Nitrospirae*, and *WS3*. Thus we confirmed the importance of these two soil variables in regulating the OJ rhizosphere. In contrast, total N, NO_3_^−^-N, and NH_4_^+^-N concentration affect bacterial assemblages in LP soil. A strong relationship between soil physicochemical properties and bacterial communities was also observed in studies of water-limited soils [51]. In these soils, the abundance of *Acidobacteria* correlated positively with soil NH_4_^+^-N and total P and negatively with total N and Mg^2+^, whereas *Chloroflexi* displayed the opposite trend [51].

In both control and stressed soil, host species were confirmed to influence bacterial community structures [52]. Our PCoA data shows that bacterial communities separated between OJ and LP soil. Al-tolerant maize cultivation significantly influenced the diazotroph populations [53], a result that aligns with our results with turfgrass. This may be mainly attributed to plant root exudates, which are key determinants of microbial community composition in plant-microorganism interactions [54].

## Conclusions

This study describes turfgrass growth and photosynthetic responses to shade stress and also describes the soil-plant feedback effects of shade tolerance in LP and OJ turfgrasses. Maintenance of higher photosynthetic capacity and root growth during shade stress in OJ make this a more shade-tolerant species than LP. Shade-intolerant LP was more responsive to both light and soil microbe-mediated soil-plant feedback compared to shade-tolerant OJ. This underscores the significance of plant-soil interactions in the underlying mechanism of shade tolerance in turfgrasses. It also confirms that the shade tolerance of OJ and LP is mediated by the rhizosphere soil bacterial community structure. In particular, greater abundance in genera of *Kaistobacter*, *Steroidobacter*, and *Pseudomonas* belonging to Alpha and gamma *Proteobacteria* may play a vital role in microbemediated mechanisms of OJ plant shade tolerance. This study also showed that under shade stress, some soil properties showed a tight coupling with several major bacterial communities, indicating that they are important drivers determining bacterial community structures. This work assists the development of strategies to combat shade in turf grass management and supports future studies of plant-microbe interaction under shade stress in turf grasses.

## Methods

### Glasshouse experimental setup and soil sampling

A glasshouse experiment was conducted at Northwest Agriculture & Forestry University, China, using soils collected from two different turfgrasses: dwarf lilyturf (*O. japonicus*) and perennial ryegrass (*L. perenne* cvs. ph.D.). These two species were grown in the same lawn but with different light environment. The seeds were obtained in June 2017, from Bcyseed Co., Ltd., Liwan District (23.072127 N, 113.207089E), Guangzhou, China. The specimen was purchased by Bcyseed Co., Ltd., a professional seed production and sales company, who undertook the formal identification of the seeds used in this study. Based on the unlikeliness of an erroneous identification, we have not deposited a voucher specimen. In this study, dwarf lilyturf (OJ) and perennial ryegrass (LP) were planted around the university campus on the same lawn: OJ (shade-tolerant) was grown around buildings and trees and LP (shade-intolerant) was planted in the center of the lawn that served as a greening square with good light conditions. The site has a warm temperate continental monsoon climate, with a mean annual air temperature of 12 °C and 500 mm of mean annual precipitation. Before the experiment, the sites were managed as turfgrass for over 10 years.

To understand the contrasting physiological mechanisms of shade tolerance between OJ and LP, we investigated the responses of plant root growth and photosynthetic capacity to shade stress. The two plant types were cultured in a plastic pot (13.5 × 17.5 × 11.0 cm) filled with soil collected from their respective areas of growth, as described above. Seeds were superficially disinfected with 0.1% sodium hypochlorite and washed three times with purified water. Three seeds were sown per pot directly into the soil. The plants were maintained in a greenhouse with an average temperature of 23/18 °C (day/night), 700 μmol m^− 2^ s^− 1^ photosynthetic active radiation from natural sunlight, and 65% relative humidity until the plants grew above 15 cm. Shade treatments were performed under the canopy using two layers of black nylon net using the same conditions described above. Pot treatments were randomized within a glasshouse compartment. We carried out five treatments including: (1) Control (0 d); (2) shade for 7 d; (3) shade for 14 d; (4) light (sunny) for 7 d; (5) light (sunny) for 14 d. There were six replicates per treatment, giving a total of 60 pots. After shade treatment, total leaf area, root morphology, chlorophyll content and the maximum quantum yield of PSII (*F*_*v*_*/F*_*m*_) were measured.

To further clarify whether soil bacterial communities directly or indirectly influenced the shade tolerance, we carried out a soil-plant feedback experiment. Soil in the 0–15 cm soil layer was collected beneath OJ and LP turfgrasses grown the same lawn as the first experiment. After the soil from under each plant type was pooled and homogenized, half of the soil was kept at 4 °C whereas the remainder was steam-sterilized for 3 h at 121 °C and then kept at 4 °C until the start of the experiment [26].

A split-plot design with 12 blocks was applied in the soil-plant feedback experiment [55]. Light treatments (sunlight or shade) were established on whole blocks. Treatments of soil biota (live, sterile) and species (OJ and LP) were set up within blocks. Each treatment combination was replicated six times, resulting in a total of 48 pots. Seeds were surface-sterilized and germinated in plug trays filled with sterilized sands until the plants grew above 3 cm. The seedlings were transplanted into pots filled with a mix of sterilized sand and either sterilized or live soil collected from beneath OJ and LP turfgrasses (sand: soil volume ratio of 9: 1).

The seedlings were placed in the greenhouse for eight weeks, during which time they were irrigated twice weekly and monitored for various parameters. Two weeks after transplanting, Hoagland solution the was supplied to each pot. Pots were randomly located in the greenhouse and re-arranged weekly to avoid possible positioning effects. Shade treatment was performed on eight-week old seedlings using two layers of black nylon net in the same greenhouse conditions as the first experiment. After four weeks of shade treatment, the seedlings’ dry biomass was analyzed.

To further investigate soil bacterial communities and the difference between OJ and LP plants, we further analyzed the effects of shade stress on their rhizospheric soil bacterial communities. The rhizosphere soil was collected from beneath OJ and LP turf grasses treated as described in the above-mentioned plant growth and photosynthetic response experiment. The roots of each plant were separated from the soil and shaken manually to remove the loosely attached soil. Rhizosphere soil was considered soil that adhered to the roots and this was collected [53]. A single rhizosphere soil sample was obtained by pooling soil from three plants growing in the same pot. As a result, each treatment had six replicate rhizosphere samples due to the six replicate pots. Bulk soil samples were also collected from the same point at a depth of 0–10 cm. Soil samples were mixed thoroughly, divided into two parts, stored in sterile 50 mL Falcon tubes, and transported to the laboratory. One part was kept at 4 °C for analysis of soil NH_4_^+^-N and NO_3_^−^-N, and to extract soil DNA within 3 days. The other part was air-dried for measurements of soil pH, total N (TN), total P (TP), total K (TK), soil organic C (SOC), available P (AP), available K (AK).

### Plant sampling measurements

The total leaf area for each seedling was measured in the laboratory using a LI-3000A leaf area scanner (LI-COR Inc., USA). Root morphology including total root length, root surface area, and root volume was analyzed using a WinRhizo-V700 root scanner (Regent Instruments Inc., Quebec, Canada). The chlorophyll content was determined spectrophotometrically using 80% acetone as a solvent [56]. On the same leaf, a portable pulse-modulated fluorometer (PAM2100, Walz, Effeltrich, Germany) with the PamWin software was used to measure chlorophyll fluorescence (*F*_*v*_*/F*_*m*_). The seedling biomass was determined by oven drying at 80 °C for 72 h. Specific leaf area (the one-sided area of a fresh leaf divided by its oven-dry weight) and specific root length (the ratio of root length to dry weight of fine roots) were measured following standard protocols [57]. N concentration in leaves and roots was measured using an elemental analyzer system (ECS 4024, Costech Inc., Italy). Plant shade response index and soil-plant feedback index were calculated based on plant biomass in shade and non-shade [18] or live and sterile soil [58].

### Soil physicochemical analyses

Soil pH was measured using a pH meter (Mettler Toledo FE20, Switzerland) in a soil solution with a 1:2.5 soil: water ratio. The NH_4_^+^-N and NO_3_^−^-N were extracted with 2.0 M KCl and measured by a continuous flow analyzer (Flowsys, Systea Inc., Italy). Soil was processed for C content by first removing inorganic C through treatment with 1 M HCl. Following removal of inorganic C, soil organic C was analyzed using an auto-analyzer (Shimadzu, Kyoto, Japan). The total N in the soils was measured on an elemental analyzer (ECS 4024, Costech Inc., Italy). Total P was determined by digesting samples first with HClO_4_-H_2_SO_4_, and then quantitated using the molybdenum blue method. An ultraviolet-visible spectrophotometer was used for this quantitation (UV-1000, AOE Instruments, Shanghai, China). Available soil P (AP) was extracted with 0.03 M ammonium fluoride-hydrochloric acid and measured colorimetrically as described above. Total K was determined using NaOH fusion method, and the available K (AK) was extracted with 1.0 M ammonium acetate and measured by flame photometry (Model 410, Sherwood, England).

### Soil bacterial community analyses

#### DNA extraction, PCR amplification, and high-throughput sequencing

Total soil DNA was extracted from 0.30 g of each soil sample using the Power Soil DNA extraction kit (MoBio Laboratories, Carlsbad, CA) as directed by the manufacturer’s instructions. The following PCR primers were used for amplification targeting the V4 region of the bacterial 16S rRNA gene: F515 (5′-GTGCCAGCMGCCGCGGTAA-3′) and R806 (5′-GGACTACHVGGGTWTCTAAT-3′). Paired-end sequencing was performed at Beijing Genomics Institute (BGI)-Shenzhen, Shenzhen, China, using a paired 250-bp Illumina HiSeq 2500 sequencing platform according to the manufacturer’s instructions.

#### Sequence processing

Illumina sequencing data were pair-assembled using FLASH software (v1.2.11) [59] with a minimal overlap length of 15 bp and mismatching ratio of overlapped region ≤0.1. Sequences were clustered into operational taxonomic units (OTUs) at a 97% identity threshold using USEARCH (v7.0.1090) [60]. UCHIME (v4.2.40) against the SILVA database to filter out chimeric sequences. USEARCH GLOBAL was used to align representative sequences from individual OTUs [61]. These were taxonomically classified using the Ribosomal Database Project (RDP) Classifier v.2.2 based on the SILVA database, using a 0.6 confidence value cutoff.

### Statistical analyses

Analysis of variance (ANOVA) according to the general linear model procedure of SPSS17.0 (SPSS Inc., Chicago, IL USA) was used to analyze the effects of shade treatment, turfgrass species, and the interactions between these factors on plant physiological indicators and the influence of shade treatment on soil properties. Two-way ANOVA was used to analyze the roles of shade stress, soil treatment and the interactions of shade stress and soil treatment in plant biomass, root: shoot ratios, and N content in OJ and LP seedlings. Values for the plant shade response index were also analyzed using two-way ANOVA, with soil treatments and species identity as the fixed factors. In addition, the soil-plant feedback index and dry biomass in OJ and LP were analyzed using two-way ANOVA, with light treatments and species identity as the fixed factors. Differences between treatment means were separated by Fisher’s protected least significance difference (LSD) test at *P =* 0.05. For these analyses, OTUs defined at 97% sequence similarity were used. Boxplots and heatmaps were obtained with the R package ggplot2 (v2.2.1). Rarefaction curves of observed OTUs were generated using R (v3.1.1). The differences in OTU composition between samples were displayed using principal component analysis (PCA). Alpha diversity [Richness, Shannon diversity index (H′) and Simpson’s Evenness (E)] was analyzed based on randomly rarefied OTU abundance matrices using mothur (v1.31.2). Bray-Curtis distances of bacterial communities using QIIME (v1.80) were used to analyze beta diversity. Principal coordinates analyses (PCoA), based on Bray-Curtis dissimilarity, were used to display differences in the composition of bacterial communities between OJ and LP rhizosphere soil treatments. Permutational multivariate analysis of variance (PERMANOVA) was conducted to test the significance of the Bray-Curtis dissimilarity. Kruskal-Wallis tests were performed using R software (kruskal. Test function) to assess the impact of shade stress on soil bacterial community structure in both species. A value of *P* < 0.05 was considered to be statistically significant.

To analyze the correlations between soil physicochemical parameters and bacterial community compositions, a Mantel test (9999 permutations) with Spearman correlations of the R vegan package was used. Canonical correspondence analyses (CCA) were performed with the R vegan package (v2.4.2) to visualize the relationship between soil physicochemical properties and bacterial communities. For the CCA analyses, the correlation of the canonical axes with the explanatory matrix was determined with the general permutation test and the “envfit” function was used to analyze the significance of soil physicochemical factors on the composition of bacterial communities. To analyze the correlations between above and below ground phenotypes and the composition of bacterial communities, pairwise fitting analysis was carried out using the “lm” function in the R vegan package.

Indicator species analysis was performed using the *multipatt* function implemented in the indicspecies package in R with 1000 permutations. The bioindicators of LP and OJ soil were designated as the OTUs of the core microbiome of LP or OJ soil under different treatments while also having abundances higher in OJ according to the permutation test (*P* < 0.05).

## Supplementary information

**Additional file 1: Table S1.** Two-way ANOVA for the effect of shade stress on morphology and photosynthesis in shade-tolerant OJ (*Ophiopogon japonicus*) and shade-intolerant LP (*Lolium perenne*) plant. LA, Total leaf area; RL, Total root length; RV, Root volume; RSA, Root surface area; Chl, Chlorophyll. ***P < 0.001, **P < 0.01, *P < 0.05.

**Additional file 2: Table S2.** Two-way ANOVA for the effect of shade stress on total plant biomass, shoot biomass, root:shoot ratios, specific root length, specific leaf area, and leaf and root N content in shade-tolerant OJ (*Ophiopogon japonicus*) and shade-intolerant LP (*Lolium perenne*) plant. Data indicated F values calculated by ANOVA. ***P < 0.001, **P < 0.01, *P < 0.05.

**Additional file 3: Table S3**. Two-way ANOVA for plant shade response index in live or sterile soil treatments. Data indicated F values calculated by ANOVA. ***P < 0.001, **P < 0.01, *P < 0.05.

**Additional file 4: Figure S1.** Rarefaction curve of bacterial 16S rRNA gene sequences obtained from amplicon sequencing.

**Additional file 5: Figure S2.** Principal Component Analysis (PCA) in rhizosphere soil microbial communities of shade-tolerant OJ (*Ophiopogon japonicus*) and shade-intolerant LP (*Lolium perenne*) under shade stress. OTUs delimited at 97% similarity.

**Additional file 6: Table S4.** List of the OTUs which comprise the core bacteriome of rhizosphere soil in OJ and LP. Those OTUs which are members of each core bacteriome are indicated in grey. Indicator species analysis was performed using the *multipatt* function implemented in the indicspecies package in R with 1000 permutations. Significance of each indicator value is represented: *P < 0.05; **P < 0.01 and ***P < 0.001.

**Additional file 7: Table S5.** Spearman’s rank correlation coefficient of soil physicochemical variables and bacterial community composition revealed by Mantel tests (*r* and *p* values).

**Additional file 8: Table S6.** Relationship of single soil variable (OJ/LP) and microbial taxa according to CCA analysis (*r*^*2*^ and *p* values). ***P < 0.001, **P < 0.01, *P < 0.05.

## Data Availability

All sequence files described in this paper have been submitted to the National Center for Biotechnology Information (NCBI) Sequence Read Archive (SRA) database (accession number: SRP154594).

## Abbreviations

AK: Available K
AP: Available P
CCA: Canonical correspondence analyses
LP: *Lolium perenne*
OJ: *Ophiopogon japonicus*
OTUs: Operational taxonomic units
PCoA: Principal coordinates analyses
PERMANOVA: Permutational multivariate analysis of variance
RDP: Ribosomal Database Project
SOC: Soil organic C
TK: Total K
TN: Total N
TP: Total P

## References

[1] Vialet-Chabrand S, Matthews JSA, Simkin AJ, Raines CA, Lawson T (2017). Importance of fluctuations in light on plant photosynthetic acclimation. Plant Physiol..

[2] Jiang CD, Wang X, Gao HY, Shi L, Chow WS (2011). Systemic regulation of leaf anatomical structure, photosynthetic performance, and high-light tolerance in sorghum. Plant Physiol..

[3] Fu JJ, Chu XT, Sun YF, Yang LY, Xu YF, Hu TM (2014). Exogenously applied nitric oxide (NO) alleviates shade-induced oxidative stress in tall fescue. J Hortic Sci Biotechnol..

[4] Mishanin VI, Trubitsin BV, Benkov MA, Minin AA, Tikhonov AN (2016). Light acclimation of shade-tolerant and light-resistant *Tradescantia* species: induction of chlorophyll *a* fluorescence and P_700_ photooxidation, expression of PsbS and Lhcb1 proteins. Photosynth Res..

[5] Li L, Li XY, Xu XW, Lin LS, Zeng FJ, Chen FL (2014). Assimilative branches and leaves of the desert plant *Alhagi sparsifolia* Shap. possesses a different adaptation mechanism to shade. Plant Physiol Biochem..

[6] Jiang YW, Duncan RR, Carrow RN (2004). Assessment of low light tolerance of seashore paspalum and bermudagrass. Crop Sci..

[7] Xu YF, Sun XL, Jin JW, Zhou H (2010). Protective effect of nitric oxide on light-induced oxidative damage in leaves of tall fescue. J Plant Physiol..

[8] Ciolfi A, Sessa G, Sassi M, Possenti M, Salvucci S, Carabelli M, Morelli G, Ruberti I (2013). Dynamics of the shade-avoidance response in Arabidopsis. Plant Physiol..

[9] Kuehne C, Nosko P, Horwath T, Bauhus J (2014). A comparative study of physiological and morphological seedling traits associated with shade tolerance in introduced red oak (*Quercus rubra*) and native hardwood tree species in southwestern Germany. Tree Physiol..

[10] Koh KJ, Bell GE, Martin DL (2003). Shade and airflow restriction effects on creeping bentgrass golf greens. Crop Sci..

[11] Hussain S, Iqbal N, Brestic M (2019). Changes in morphology, chlorophyll fluorescence performance and Rubisco activity of soybean in response to foliar application of ionic titanium under normal light and shade environment. Sci Total Enviro..

[12] Xi NX, Bloor JMG, Wang Y, Chu CJ (2019). Contribution of conspecific soil microorganisms to tree seedling light responses: Insights from two tropical species with contrasting shade tolerance. Environ Exp Bot.

[13] Valladares F, Niinemets U (2008). Shade tolerance, a key plant feature of complex nature and consequences. Annu Rev Ecol Evol S..

[14] Bonfls CJW, Phillips TJ, Lawrence DM, Cameron-Smith P, Riley WJ, Subin ZM (2012). On the infuence of shrub height and expansion on northern high latitude climate. Environ Res Lett..

[15] Friesen ML, Porter SS, Stark SC, von Wettberg EJ, Sachs JL, Martinez-Romero E (2011). Microbially mediated plant functional traits. Ann Rev Ecol Evol Syst..

[16] Fry E, Johnson G, Hall A, Pritchard W, Bullock J, Bardgett R (2018). Drought neutralises plant-soil feedback of two mesic grassland forbs. Oecologia..

[17] Berg G, Rybakova D, Grube M, Köberl M (2016). The plant microbiome explored: implications for experimental botany. J Exp Bot..

[18] Bruelheide H, Dengler J, Purschke O, Lenoir J, Jiménez-Alfaro B, Hennekens SM, Botta-Dukát Z, Chytrý M, Field R, Jansen F, Kattge J, Pillar VD, Schrodt F, Mahecha MD, Peet RK, Sandel B, van Bodegom P, Altman J, Alvarez-Dávila E, MAS AK, Attorre F, Aubin I, Baraloto C, Barroso JG, Bauters M, Bergmeier E, Biurrun I, Bjorkman AD, Blonder B, Čarni A, Cayuela L, Černý T, JHC C, Craven D, Dainese M, Derroire G, De Sanctis M, Díaz S, Doležal J, Farfan-Rios W, Feldpausch TR, Fenton NJ, Garnier E, Guerin GR, Gutiérrez AG, Haider S, Hattab T, Henry G, Hérault B, Higuchi P, Hölzel N, Homeier J, Jentsch A, Jürgens N, Kącki Z, Karger DN, Kessler M, Kleyer M, Knollová I, Korolyuk AY, Kühn I, Laughlin DC, Lens F, Loos J, Louault F, Lyubenova MI, Malhi Y, Marcenò C, Mencuccini M, Müller JV, Munzinger J, Myers-Smith IH, Neill DA, Niinemets Ü, Orwin KH, Ozinga WA, Penuelas J, Pérez-Haase A, Petřík P, Phillips OL, Pärtel M, Reich PB, Römermann C, Rodrigues AV, Sabatini FM, Sardans J, Schmidt M, Seidler G, Silva Espejo JE, Silveira M, Smyth A, Sporbert M, Svenning JC, Tang Z, Thomas R, Tsiripidis I, Vassilev K, Violle C, Virtanen R, Weiher E, Welk E, Wesche K, Winter M, Wirth C, Jandt U (2018). Global trait-environment relationships of plant communities. Nat Ecol Evol.

[19] Dahl MB, Priemé A, Brejnrod A, Brusvang P, Lund M, Nymand J, Kramshøj M, Ro-Poulsen H, Haugwitz MS (2017). Warming, shading and a moth outbreak reduce tundra carbon sink strength dramatically by changing plant cover and soil microbial activity. Sci Rep..

[20] Hartlea RT, Fernandez GCJ, Nowaka RS (2006). Horizontal and vertical zones of influence for root systems of four Mojave Desert shrubs. J Arid Environ..

[21] Pérez-Jaramillo JE, Carrión VJ, Bosse M, Ferrão LFV, de Hollander M, Garcia AAF, Ramírez CA, Mendes R, Raaijmakers JM (2017). Linking rhizosphere microbiome composition of wild and domesticated *Phaseolus vulgaris* to genotypic and root phenotypic traits. ISME J.

[22] Saleem M, Law AD, Moe LA (2016). Nicotiana roots recruit rare rhizosphere taxa as major root-inhabiting microbes. Microb Ecol.

[23] Saleem M, Law AD, Sahib MR, Pervaizc ZH, Zhang QM (2018). Impact of root system architecture on rhizosphere and root microbiome. Rhizosphere.

[24] Ma ZY, Behling S, Ford ED (2014). The contribution of dynamic changes in photosynthesis to shade tolerance of two conifer species. Tree Physiol..

[25] Ploschuk EL, Bado LA, Salinas M, Wassner DF, Windauer LB, Insausti P (2014). Photosynthesis and fluorescence responses of Jatropha curcas to chilling and freezing stress during early vegetative stages. Environ Exp Bot..

[26] Rutten G, Prati D, Hemp A, Fischer M (2016). Plant-soil feedback in East-African savanna trees. Ecology..

[27] Xi NX, Chu CJ, Bloor JMG (2018). Plant drought resistance is mediated by soil microbial community structure and soil-plant feedbacks in a savanna tree species. Environ Exp Bot..

[28] Wardle DA, Bardgett RD, Klironomos JN, Setala H, van der Putten WH, Wall DH (2004). Ecological linkages between aboveground and belowground biota. Sci..

[29] Kobe RK, Vriesendorp CF (2011). Conspecific density dependence in seedlings varies with species shade tolerance in a wet tropical forest. Ecol Lett..

[30] Bloom AJ, Chapin FS, Mooney HA (1985). Resource limitation in plants-an economic analogy. Ann Rev Ecol Syst..

[31] Acosta-Martínez V, Cotton J, Gardner T, Moore-Kucera J, Zak J, Wester D, Cox S (2014). Predominant bacterial and fungal assemblages in agricultural soils during a record drought/heat wave and linkages to enzyme activities of biogeochemical cycling. Appl Soil Ecol..

[32] Tóth Z, Táncsics A, Kriszt B, Kröel-Dulay G, Ónodi G, Hornung E (2017). Extreme effects of drought on composition of the soil bacterial community and decomposition of plant tissue: bacterial community and plant tissue decomposition. Eur J Soil Sci..

[33] Ikenaga M, Guevara R, Dean AL, Pisani C, Boyer JN (2010). Changes in community structure of sediment bacteria along the Florida coastal Everglades marsh-mangrove-seagrass salinity gradient. Microb Ecol..

[34] Yuste JC, Fernandez-Gonzalez AJ, Fernandez-Lopez M, Ogaya R, Penuelas J, Sardans J, Lloret F (2014). Strong functional stability of soil microbial communities under semiarid Mediterranean conditions and subjected to long-term shifts in baseline precipitation. Soil Biol Biochem..

[35] Van Horn DJ, Okie JG, Buelow HN, Gooseff MN, Barrett JE, Takacs-Vesbacha CD (2014). Soil microbial responses to increased moisture and organic resources along a salinity gradient in a Polar Desert. Appl Environ Microb..

[36] Hartmann M, Brunner I, Hagedorn F, Bardgett RD, Stierli B, Herzog C, Chen XM, Zingg A, Grafpannatier E, Rigling A, Frey B (2017). A decade of irrigation transforms the soil microbiome of a semi-arid pine forest. Mol Ecol..

[37] Frossard A, Donhauser J, Mestrot A, Gygax S, Bååth E, Frey B (2018). Long- and short-term effects of mercury pollution on the soil microbiome. Soil Biol Biochem..

[38] Yandigeri MS, Meena KK, Singh D, Malviya N, Singh DP, Solanki MK, Yadav AK, Arora DK (2012). Drought-tolerant endophytic actinobacteria promote growth of wheat (*Triticum aestivum*) under water stress conditions. Plant Growth Regul..

[39] Singh BK, Munro S, Potts JM, Millard P (2007). Influence of grass species and soil type on rhizosphere microbial community structure in grassland soils. Appl Soil Ecol..

[40] Chodak M, Gołębiewski M, Morawska-Płoskonka J, Kuduk K, Niklińska M (2015). Soil chemical properties affect the reaction of forest soil bacteria to drought and rewetting stress. Ann Microbiol..

[41] Dimkpa C, Weinand T, Asch F (2009). Plant–rhizobacteria interactions alleviate abiotic stress conditions. Plant Cell Environ..

[42] Kaushal M, Wani SP (2016). Plant-growth-promoting rhizobacteria: drought stress alleviators to ameliorate crop production in drylands. Ann Microbiol..

[43] Kumar M, Mishra S, Dixit V, Kumar M, Agarwal L, Chauhan PS, Nautiyal CS (2016). Synergistic effect of *Pseudomonas putida* and *Bacillus amyloliquefaciens* ameliorates drought stress in chickpea (*Cicer arietinum* L.). Plant Signal Behav.

[44] Etesami H, Maheshwari DK (2018). Use of plant growth promoting rhizobacteria (PGPRs) with multiple plant growth promoting traits in stress agriculture: Action mechanisms and future prospects. Ecotox Environ Safe..

[45] Bouskill NJ, Lim HC, Borglin S, Salve R, Wood TE, Silver WL, Brodie EL (2013). Pre-exposure to drought increases the resistance of tropical forest soil bacterial communities to extended drought. ISME J..

[46] Ling N, Chen D, Guo H, Wei JX, Bai YF, Shen QR, Hu SJ (2017). Differential responses of soil bacterial communities to long-term N and P inputs in a semi-arid steppe. Geoderma..

[47] Pascual J, Blanco S, Ramos JL, van Dillewijn P (2018). Responses of bulk and rhizosphere soil microbial communities to thermoclimatic changes in a Mediterranean ecosystem. Soil Biol Biochem..

[48] Bottomley PJ, Taylor AE, Myrold DD (2012). A consideration of the relative contributions of different microbial subpopulations to the soil N cycle. Front Microbiol..

[49] Boyle SA, Yarwood RR, Bottomley PJ, Myrold DD (2008). Bacterial and fungal contributions to soil nitrogen cycling under Douglas fir and red alder at two sites in Oregon. Soil Biol Biochem..

[50] Nguyen LTT, Osanai Y, Lai KT, Anderson IC, Bange MP, Tissue DT, Singh BK (2018). Responses of the soil microbial community to nitrogen fertilizer regimes and historical exposure to extreme weather events: Flooding or prolonged-drought. Soil Biol Biochem..

[51] Bachar A, Al-Ashhab A, Soares MIM, Sklarz MY, Angel R, Ungar ED, Gillor O (2010). Soil microbial abundance and diversity along a low precipitation gradient. Microb Ecol..

[52] Naylor D, DeGraaf S, Purdom E, Coleman-Derr D (2017). Drought and host selection influence bacterial community dynamics in the grass root microbiome. ISME J..

[53] Wang C, Zheng MM, Hu AY, Zhu CQ, Shen RF (2018). Diazotroph abundance and community composition in an acidic soil in response to aluminum-tolerant and aluminum-sensitive maize (*Zea mays* L.) cultivars under two nitrogen fertilizer forms. Plant Soil..

[54] Li B, Li YY, Wu HM, Zhang FF, Li CJ, Li XX, Lambers H, Li L (2016). Root exudates drive interspecific facilitation by enhancing nodulation and N_2_ fixation. P Natl Acad Sci USA..

[55] Quinn GP, Keough MJ (2002). Experimental design and data analysis for biologists.

[56] Lichtenthaler HK (1987). Chlorophylls and carotenoids: Pigments of photosynthetic biomembranes. Method Enzymol..

[57] Pérez-Harguindeguy N, Díaz S, Garnier E, Lavorel S, Poorter H, Jaureguiberry P, Bret-Harte MS, Cornwell WK, Craine JM, Gurvich DE, Urcelay C, Veneklaas EJ, Reich PB, Poorter L, Wright IJ, Ray P, Enrico L, Pausas JG, de Vos AC, Buchmann N, Funes G, Quétier F, Hodgson JG, Thompson K, Morgan HD, ter Steege H, Sack L, Blonder B, Poschlod P, Vaieretti MV, Conti G, Staver AC, Aquino S, JHC C (2013). New handbook for standardised measurement of plant functional traits worldwide. Aust J Bot.

[58] Brinkman E, Van der Putten WH, Bakker EJ, Verhoeven KJF (2010). Plant-soil feedback: experimental approaches, statistical analyses and ecological interpretations. J Ecol..

[59] Magoc T, Salzberg S (2011). FLASH: Fast length adjustment of short reads to improve genome assemblies. Bioinformatics..

[60] Edgar RC (2013). UPARSE: Highly accurate OTU sequences from microbial amplicon reads. Nat Methods..

[61] Wang Q, Garrity GM, Tiedje JM, Cole JR (2007). Naive Bayesian classifier for rapid assignment of rRNA sequences into the new bacterial taxonomy. Appl Environ Microb..

