# Supplementary material for: Effects of shade stress on turfgrasses morphophysiology and rhizosphere soil bacterial communities
Source: BMC Plant Biol. 2020 Mar 2;20:92. doi: 10.1186/s12870-020-2300-2 (PMC7053125; doi:10.1186/s12870-020-2300-2)
Supplement: Supplementary file 4 — Additional file 4: Figure S1. Rarefaction curve of bacterial 16S rRNA gene sequences obtained from amplicon sequencing. [file 12870_2020_2300_MOESM4_ESM.pdf]

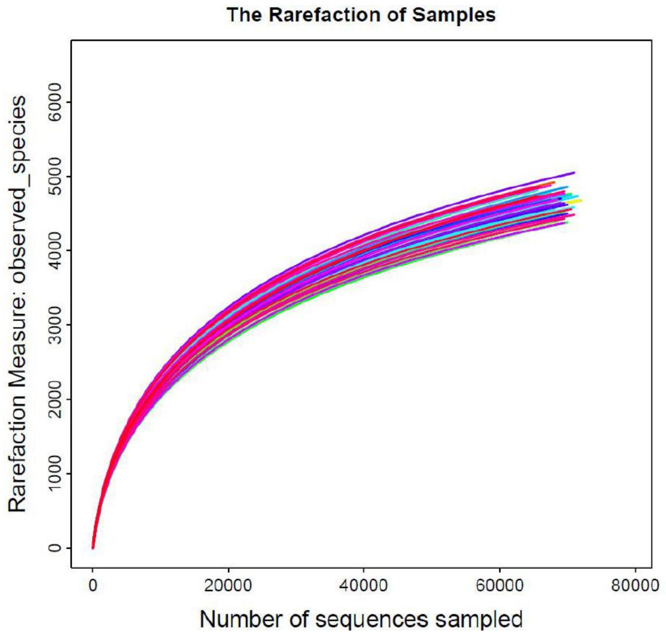

Figure S1. Rarefaction curve of bacterial 16S rRNA gene sequences obtained from amplicon sequencing.
