# Supplementary material for: Effects of shade stress on turfgrasses morphophysiology and rhizosphere soil bacterial communities
Source: BMC Plant Biol. 2020 Mar 2;20:92. doi: 10.1186/s12870-020-2300-2 (PMC7053125; doi:10.1186/s12870-020-2300-2)
Supplement: Supplementary file 5 — Additional file 5: Figure S2. Principal Component Analysis (PCA) in rhizosphere soil microbial communities of shade-tolerant OJ (Ophiopogon japonicus) and shade-intolerant LP (Lolium perenne) under shade stress. OTUs delimited at 97% similarity. [file 12870_2020_2300_MOESM5_ESM.pdf]

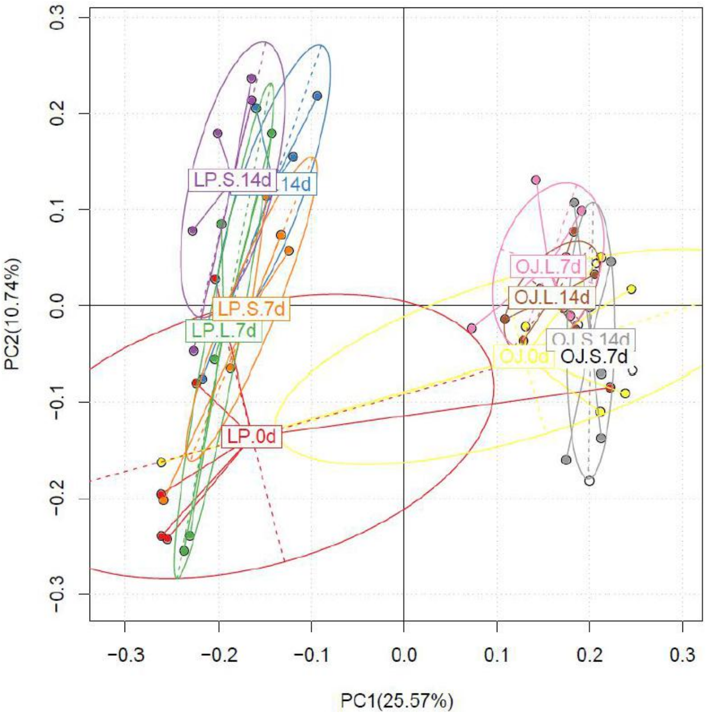

Figure S2. Principal Component Analysis (PCA) in rhizosphere soil microbial communities of shade-tolerant OJ (*Ophiopogon japonicus*) and shade-intolerant LP (*Lolium perenne*) under shade stress. OTUs delimited at 97% similarity.
